# Robustness optimization for rapid prototyping of functional artifacts based on visualized computing digital twins

**DOI:** 10.1186/s42492-023-00131-w

**Published:** 2023-02-27

**Authors:** Jinghua Xu, Kunqian Liu, Linxuan Wang, Hongshuai Guo, Jiangtao Zhan, Xiaojian Liu, Shuyou Zhang, Jianrong Tan

**Affiliations:** 1 State Key Lab of Fluid Power and Mechatronic Systems, Zhejiang University, Hangzhou 310058, China; 2 Key Lab of Advanced Manufacturing Technology of Zhejiang Province, Zhejiang University, Hangzhou 310058, China; 3 Engineering Research Center for Design Engineering and Digital Twin of Zhejiang Province, Zhejiang University, Hangzhou 310058, China; 4 Zhejiang-Singapore Innovation and AI Joint Research Lab, Zhejiang University, Hangzhou 310058, China; 5 School of Creative Arts and design, Zhejiang Institute of Mechanical and Electrical Engineering, Hangzhou 310053, China; 6 Ningbo Research Institute, Zhejiang University, Ningbo 315100, China

**Keywords:** Robustness optimization design, Rapid prototyping, Functional artifacts, Fuzzy decision-making, Infrared thermographs, Visualized computing digital twins

## Abstract

This study presents a robustness optimization method for rapid prototyping (RP) of functional artifacts based on visualized computing digital twins (VCDT). A generalized multiobjective robustness optimization model for RP of scheme design prototype was first built, where thermal, structural, and multidisciplinary knowledge could be integrated for visualization. To implement visualized computing, the membership function of fuzzy decision-making was optimized using a genetic algorithm. Transient thermodynamic, structural statics, and flow field analyses were conducted, especially for glass fiber composite materials, which have the characteristics of high strength, corrosion resistance, temperature resistance, dimensional stability, and electrical insulation. An electrothermal experiment was performed by measuring the temperature and changes in temperature during RP. Infrared thermographs were obtained using thermal field measurements to determine the temperature distribution. A numerical analysis of a lightweight ribbed ergonomic artifact is presented to illustrate the VCDT. Moreover, manufacturability was verified based on a thermal-solid coupled finite element analysis. The physical experiment and practice proved that the proposed VCDT provided a robust design paradigm for a layered RP between the steady balance of electrothermal regulation and manufacturing efficacy under hybrid uncertainties.

## Introduction

In recent years, in the field of rapid prototyping (RP), most composite materials have been formed layer-by-layer via thermal energy fields without mold patterns. Therefore, the energy distribution of parts is very important for fabrication performance [1, 2]. The study of temperature distribution is helpful in reducing heat loss, improving processing efficiency, and saving energy.

The change in the RP process puts forward higher requirements for the applicability of materials. RP includes fused deposition modeling (FDM) and fused filament fabrication. Different processing technologies have diversified the requirements for processing environments, and different printing materials have different physical properties [3, 4]. It is necessary to consider the differences in the properties of these materials when designing the printing parameters. In particular, in a thermodynamic simulation, different materials exhibit different responses to the control signal. Therefore, the rapid response to the heating signal has become a key factor affecting the processing efficiency and environmental emissions of composite material RP [5, 6].

One of the key research points concerning the improvement in the RP process performance is the realization of the RP heating temperature. The accurate control of temperature provides the basis for the reconstruction of biological tissue structures with activity [7]. Researchers [8] have studied the effect of temperature on the emission rate of microparticles in the printing process of FDM and observed that the emission rate of particles was higher at higher working temperatures.

Carbon fiber composites have new applications in 3D printing. Kuncius et al. [9] improved the FDM production technology for continuous carbon fiber composites. Hou et al. [10] proposed a 3D printing technology for continuous fiber-reinforced thermoplastic composites that controlled the content of printed fibers. Li et al. [11] replaced the traditional resistance heating method with microwave heating to achieve faster production of continuous carbon-fiber-reinforced plastic. Mohammadizadeh and Fidan [12] studied the effects of fiber parameters on the tensile properties of manufactured components. Kubota et al. [13] conducted tensile tests on samples with different stacking directions and studied the influence of the printing path.

In addition to the significant influence of the materials on the manufacturing process, the control scheme also determines the molding effect. An optimization algorithm that considers robustness can make the system operate under the interference of uncertain factors. Recently, some studies have been conducted on robust optimization in RP. The 3D printing parameters play a decisive role in printing quality. Different materials can be used as uncertainty factors to study the robustness of printing quality [14]. Material uncertainty is also used to evaluate the robustness of the optimal compliance design in additive manufacturing [15]. Naserifar et al. [16] studied the impact of 3D printing of stretchable aggregates on the robustness of wearable skin devices. Robustness is also an important factor considered in complex multiobjective optimization problems. Ehrgott et al. [17] applied robust optimization of multiobjective uncertainty to a practical field and studied the impact of weather on agricultural harvest. Gaspar-Cunha and Covas [18] used a multiobjective evolutionary algorithm to evaluate the robustness of research issues. Kotireddy et al. [19] used a genetic algorithm (GA) to improve the computational efficiency of multiobjective optimization considering uncertainty. The robustness of the design is guaranteed, and the calculation cost is reduced.

An increasing number of visualization technologies have been developed and applied for the visual presentation of digital twins (DTs), by which a physical object can be mapped into the real world and digitized in the form of software modeling. In particular, the development of DT technology makes real-time prediction, interaction, and visualization possible. Saiz et al. [20] optimized the robustness of visual defect segmentation using a generative adversarial network. Burch et al. [21] considered user interactions when implementing dynamic visualization of graphs. Fahd and Venkatraman [22] attempted to model unstructured data using visualization.

With the aim of improving printing efficiency for mixed-material 3D printing, by considering previous works [23–27] on rapid manufacturing verification of product conceptual design entities, researchers continue to investigate an approach to robust RP. Accordingly, this study proposes a robustness design method for the RP of fiber-reinforced composites based on visualized computing digital twins (VCDTs). The main difference between the well-known DT and VCDT is that the latter can integrate thermal, structural, and multidisciplinary knowledge for computed visualization.

## Methods

### Experimental methods

#### Multiobjective robustness optimization for RP

In the process of robust optimization, the randomness of the genetic operators may cause the generated individuals to fail to meet the requirements of the control model. The generated parameter combination must be robustly optimized to eliminate individuals that do not meet the constraint conditions prior to calculating the fitness function. Robustness optimization considering the uncertainty model satisfies Eq. (1).

1 $$\left\{\begin{array}{c}minf\left(X,\xi \right) \quad \forall \xi \in U \\ X={[{x}_{1},{x}_{2},\dots ,{x}_{n}]}^{T} \quad n\in {Z}^{+}\\ s.t.\ {x}_{il} \le {x}_{i} \le {x}_{iu} \\ g\left(X,\xi \right) \le 0\end{array}\right.$$

where the *X* vector can have 16 control variables, and ξ is the uncertain parameter,  *f* and *g* are objective function and constraint function respectively.

In the robust optimization of RP, the uncertain factors are the temperatures of the printing materials and environment. Upon applying a GA to fuzzy theory, the iterative population becomes more stable and efficient after considering the robustness of the system.

#### Genetic operator and population iteration of membership function

In fuzzy theory, the membership function is the key factor in determining the input signal identification and processing of the control system. This maladaptive limitation can be overcome by using a GA that can iterate the membership function. The problem of system instability introduced by random parameters can be solved by applying the theory of robust optimization to the GA. After the controlled parameter variables are determined, the appropriate fitness function is selected as the evaluation index. Considering the error *e* of each measurement and relative error *e*_c_ of the two adjacent measurements in the control process, performance index* J* is defined as shown in Eq. (2).

2 $$\small J=T\sum_{i=1}^{n}{[{e\left(i\right)}^{2}+{e_{c}\left(i\right)}^{2}]}^{1/2}$$

where *T* is the sampling time, and *n* is the number of sampling points corresponding to the parameter group. The purpose of the algorithm is to optimize the performance index *J* using the genetic theory. The randomness of parameter selection may cause the performance index to have a magnitude difference; therefore, the fitness function $F$ is more appropriate to represent the performance of each group of parameters. In contrast to the performance index $J$, the fitness function $F$ is positively correlated with the fitness of the parameter group. It is defined as follows:

3 $$F= \frac{P}{1+J}$$

where *P* is the scale coefficient, and *J* is the performance index.

**Fig. 1:**
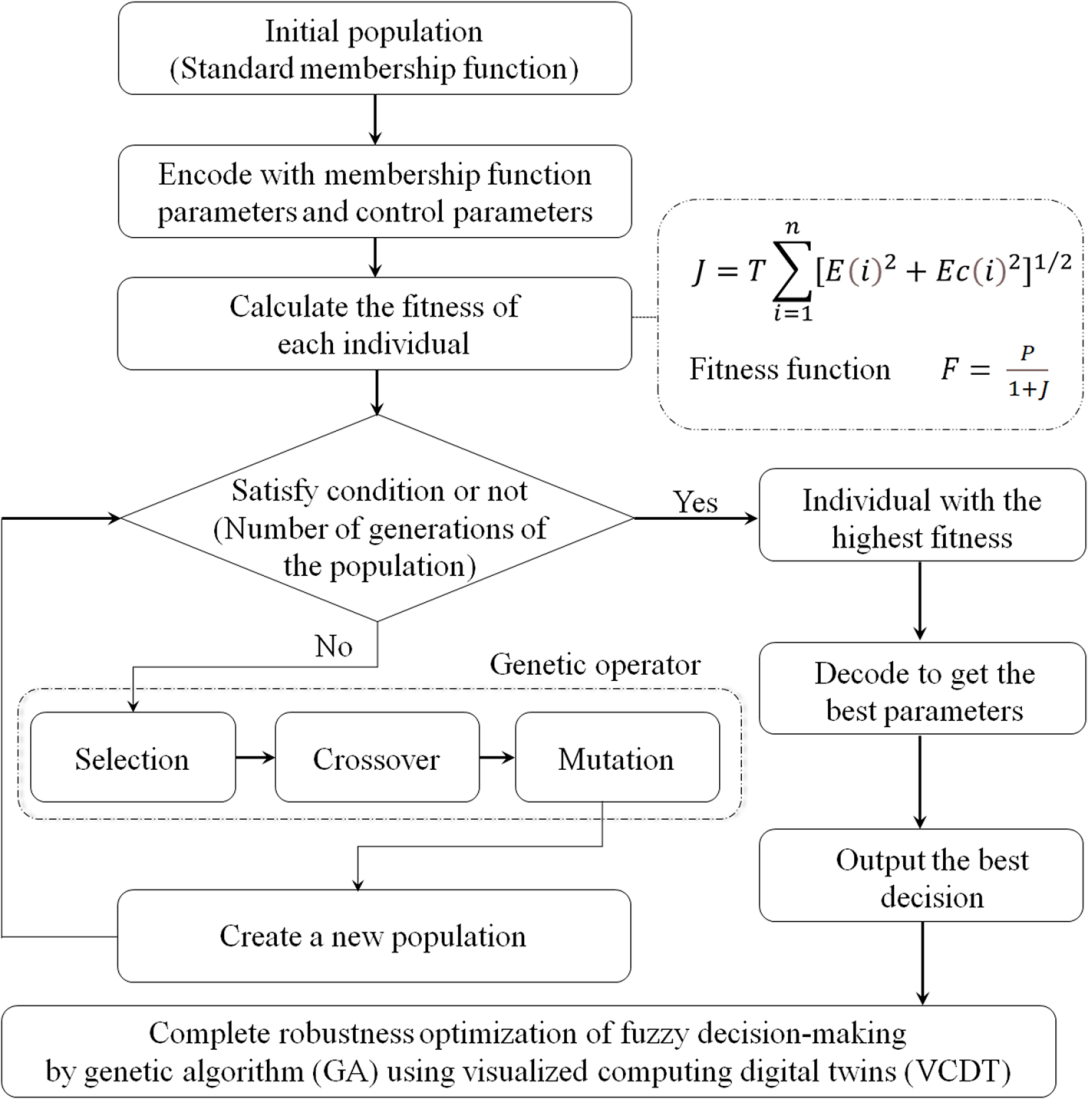
Flowchart of the membership function of fuzzy decision-making optimized by a GA

After robust control is considered, the population is iteratively optimized. In previous research [25], the membership function was defined according to expert experience. It was defined as the membership rule of initial standardization. The fitness function of the response obtained by the control algorithm based on this rule is defined as a unit value, from which the value of the proportional coefficient *P* can be determined. According to the calculations, when the proportional coefficient *P* in Eq. (3) is obtained, the default fitness function value of the initial membership function model response can be obtained, and it is determined as the value of *P*. A population with high volumes of individuals is randomly generated as the initial population, and the fitness of each individual is calculated separately. The optimal schemes are compared and converged through the selection, crossover, and mutation steps. The best individual preservation and roulette methods can be used for the selection. The two individuals with the highest fitness in each generation are directly selected to enter the next generation, and a number of individuals with higher fitness are then randomly selected to cross or mutate using this genetic operator to obtain a new population. The overall flow of the algorithm is illustrated in Fig. 1.

### Electrothermal regulating design of multimaterial RP

#### Thermodynamic equilibrium for conduction of RP

Regardless of the functional artifact *M*, axis-aligned bounding boxes (AABBs) can be generated to define the scale of the manifold structure itself. The mechanical stroke lengths ${x}_{b}{,y}_{b},{z}_{b}$ of the AABBs along the $x,y,z$ directions, respectively, can be calculated. Thus, any point $Q$ can be represented in terms of its relative position $ratio$ in the AABB.

4 $$ratio=\left(Q-\mathrm{min}(x,y,z)\right)/{[x}_{b}{,y}_{b},{z}_{b}]\quad ratio\in [\mathrm{0,1}]$$

The maximum print space for a printer along the $x,y,z$ directions are ${x}_{p}{,y}_{p},{z}_{p}$, respectively. In the printing coordinate system, the normalized height ${h}_{n}$ of the i^th^ layer can be defined as follows:

5 $${h}_{n}=\frac{{z}_{i}}{{z}_{b}}\quad { where\ h}_{n}\in (\mathrm{0,1}]$$

#### Glass fiber composite materials for functional requirements

Glass fiber (GF) is an inorganic nonmetallic material with excellent performance, which has the advantages of good insulation, strong heat resistance, good corrosion resistance, and high mechanical strength. GF is usually used as reinforcement material, electrical insulation material, or thermal insulation material in composites utilized in various manufacturing fields. GF can improve the strength and rigidity of plastics and improve their heat resistance and thermal deformation temperature. It can also improve their dimensional stability, reduce shrinkage, and reduce material deformation.

Thermal conductivity refers to the heat transferred through an area of 1 m^2^ within a certain period by a 1 m thick material with a temperature difference of 1 K on both sides of the surface under stable heat transfer conditions. The thermal conductivity *k*_*x*_ (W/(m·K)) is calculated using the following expression:

6 $${k}_{x}=-\frac{{q}_{x}^{"}}{(\partial T/\partial x)}$$

where *x* is defined as the heat flow direction, and *q*_*x*_*"* (W/m^2^) is the heat flux in this direction. ∂*T*/ ∂x (K/m) denotes the temperature gradient in the assigned direction.

The thermal conductivity of GF is considerably low. The thermal conductivity of glass is 0.7–1.28 W/(m·K). However, after being drawn into a GF, its thermal conductivity is only 0.035 W/(m·K). The main reason for this phenomenon is that the gap between the fibers is large, the density is small, and the thermal conductivity of the air in the middle is low, which reduces the thermal conductivity of the entire material. The smaller the thermal conductivity, the better the thermal insulation performance. However, when the GF is damped, the thermal conductivity increases and the thermal insulation performance decreases.

The dielectric constant is a physical parameter that characterizes the dielectric or polarization properties of a dielectric material. It is a property of the material itself and measures the ability of a material to store charge. Relative permittivity is often used to characterize the dielectric properties of a material. Relative permittivity *ε*_*r*_ is calculated by measuring the capacitance of a thin plate material using the following expression:

7 $${\varepsilon }_{r}=(C\times d)/({\varepsilon }_{0}\times S)$$

where *ε*_*r*_ is the relative dielectric coefficient of the material, *C*(*F*) is the measured capacitance, d (nm) is the sample thickness, and *S* (m^2^) is the sample area. *ε*_0_ is the vacuum dielectric constant (8.854 × 10^–12^ F/m).

The tensile strength *σ*_b_ (MPa) represents the resistance of the material to the maximum uniform plastic deformation and is the maximum stress that the material bears before breaking. Its value can be calculated using the following expression:

8 $${\sigma }_{b}={F}_{b}/{S}_{o}$$

where *F*_b_ (N) is the maximum force applied when the specimen is broken, and *S*_o_ (mm^2^) is the original cross-sectional area of the specimen.

The tensile strength of GF is significantly higher than that of glass with the same composition. For example, the tensile strength of alkali glass is only 40–100 MPa, whereas that of the GF drawn from it can reach 2000 MPa, which is 20-50 times higher. The tensile strength of GF can be even higher than that of high-strength alloy steel with the same diameter.

### Temperature fuzzy decision-making and experimental control

#### GA of proportion integration differentiation fuzzy decision-making

The improved proportion integration differentiation (PID) algorithm based on fuzzy theory can more accurately control the temperature of the system. However, the membership function defined by experience has limitations, particularly for different control models. As an excellent global search algorithm, the GA can find a global optimal solution with high efficiency. The basis of the GA optimization of fuzzy decision-making is to select appropriate parameters as genes for combination.

Several parameters were selected for variable control to simplify the genetic model. As shown in Eqs. (9) and (10), 11 key nodes in the domain of the membership function were selected as variable parameters along with input coefficients (error *e* and relative error *e*_*c*_ for each signal acquisition) and output coefficients (adjustment coefficients *K*_*p0*_, *K*_*i0*_, *K*_*d0*_ for the three factors in PID control).

9 $$f=\left\{\begin{array}{c} NB(Negative\ Big): \frac{1}{2}\left(1+\mathrm{cos}\left(\left(\frac{\pi }{{P}_{7}+6}\right)\bullet \left(x+6\right)\right)\right) -6\le x<{P}_{7}\\ NM(Negative\ Medium): \frac{x+6}{{P}_{1}+6} -6\le x<{P}_{1} ; \frac{x-{P}_{8}}{{P}_{1}-{P}_{8}} {P}_{1}\le x<{P}_{8} \\ NS(Negative\ Small): \frac{x-{P}_{6}}{{P}_{2}-{P}_{6}} {P}_{6}\le x<{P}_{2} ; \frac{x-{P}_{9}}{{P}_{2}-{P}_{9}} {P}_{2}\le x<{P}_{9}\\ Z(ZERO): \frac{x-{P}_{7}}{{P}_{3}-{P}_{7}} {P}_{7}\le x<{P}_{3} ; \frac{x-{P}_{10}}{{P}_{3}-{P}_{10}} {P}_{3}\le x<{P}_{10}\\ PS(Positive\ Small): \frac{x-{P}_{8}}{{P}_{4}-{P}_{8}} {P}_{8}\le x<{P}_{4} ; \frac{x-{P}_{11}}{{P}_{4}-{P}_{11}} {P}_{4}\le x<{P}_{11}\\ PM(Positive\ Medium): \frac{x-{P}_{9}}{{P}_{5}-{P}_{9}} {P}_{9}\le x<{P}_{5} ; \frac{x-6}{{P}_{5}-6} {P}_{5}\le x<6\\ PB(Positive\ Big): \frac{1}{2}\left(1+\mathrm{cos}\left(\left(\frac{\pi }{{P}_{10}-6}\right)\bullet \left(x-6\right)\right)\right) {P}_{10}\le x<6\end{array}\right.$$

where the range of the independent variable *x* of the membership function is [-6, 6].

10 $$\left\{\begin{array}{c}e= {P}_{12} \bullet error\\ {e}_{c}= {P}_{13}\bullet ( relative\ error)\\ {K}_{p}={K}_{p0}+{P}_{14}\bullet \left\{e,{e}_{c}\right\}\\ {K}_{i}={K}_{i0}+{P}_{15}\bullet \left\{e,{e}_{c}\right\}\\ {K}_{d}={K}_{d0}+{P}_{16}\bullet \left\{e,{e}_{c}\right\}\end{array}\right.$$

**Fig. 2:**
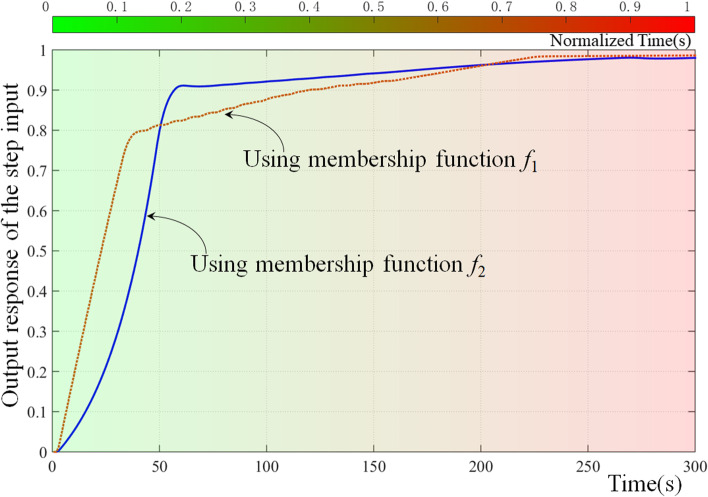
Comparison of the response curve before and after genotype change, where using membership function *f*_1_ and *f*_2_, the stable value is 0.9861 and 0.98, rise time is 108 and 55, peak time is 300 and 270 alongwith peak value 0.9861 and 0.9805, overshoot is nearly zero and 0.05%, settle time is 175 and 125

Sixteen parameters were used as gene sequences in the GA. Figure 2 shows the output response of the step input and corresponding membership function when the parameters have different values.

**Fig. 3:**
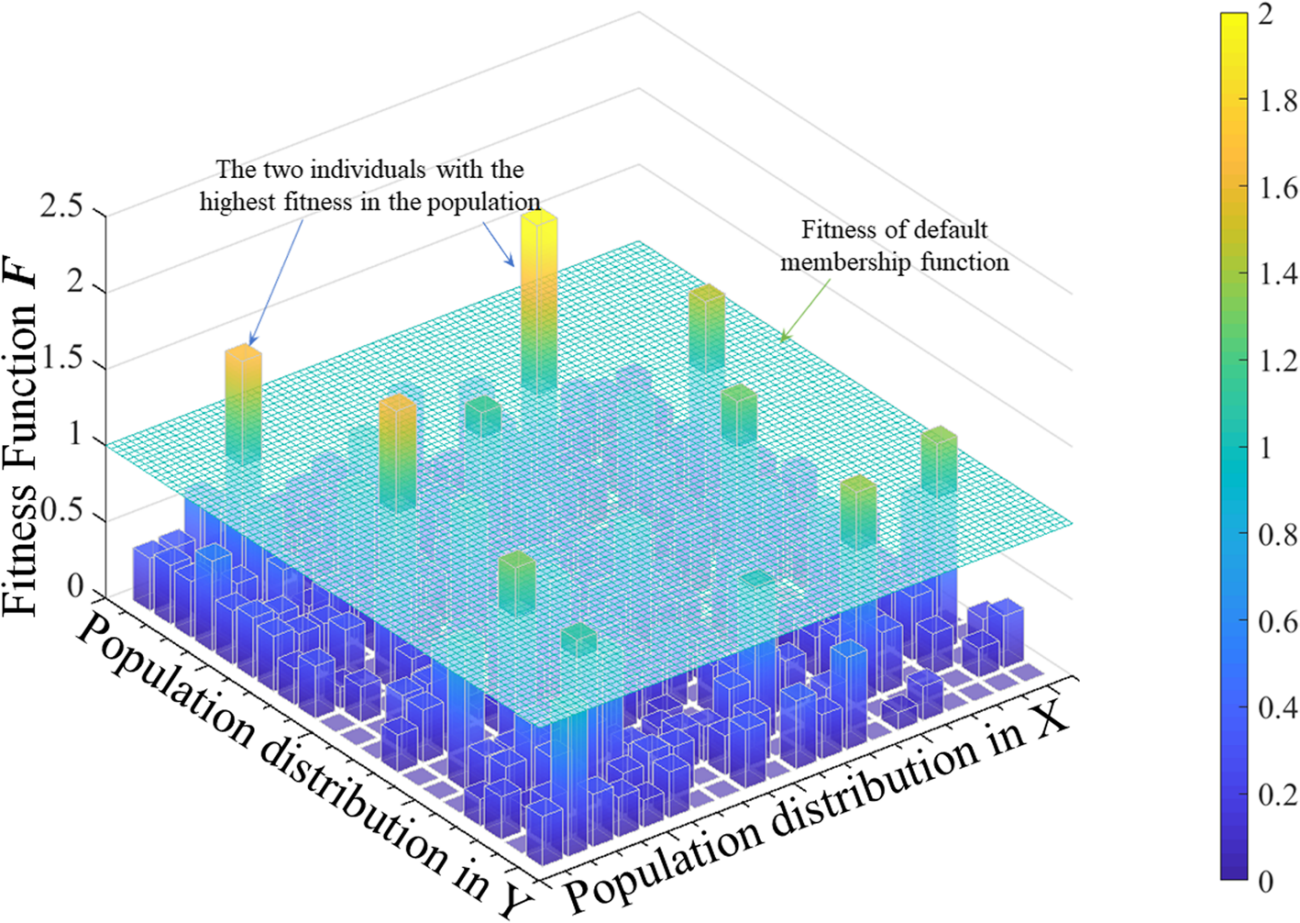
Fitness function *F* of a generation of population for fuzzy decision-making

Figure 3 shows the distribution of the intermediate-generation population. Individuals whose fitness function value is higher than the initial base level in the figure are selected for crossover and mutation to enter the next-generation population. The fitness values of the two individuals with the highest fitness function are 2.112 and 1.6706, respectively, which directly enter the next-generation population. New individuals are then generated by random coding until the number of individuals in the population reaches 400, and the fitness of the next generation is calculated and updated. After a limited number of iterations, the best individual fitness value is 2.112. This implies that the performance index *J* under the optimized membership rule is 41.1% of the initial value, significantly reducing the error and accelerating the temperature response.

#### Physical experiment of electrothermal regulating using programmable power

An actual heating experiment was conducted to further verify the effectiveness of the algorithm. The physical experiment was powered using a multichannel programmable DC linear power supply. This power supply unit can convert an input AC voltage of 220 V ± 10% at 50 Hz into DC linear power in each channel, which can be programmatically controlled independently under the mode of constant voltage, constant current, and constant resistance. Its voltage output range is 12-24 V.

**Fig. 4:**
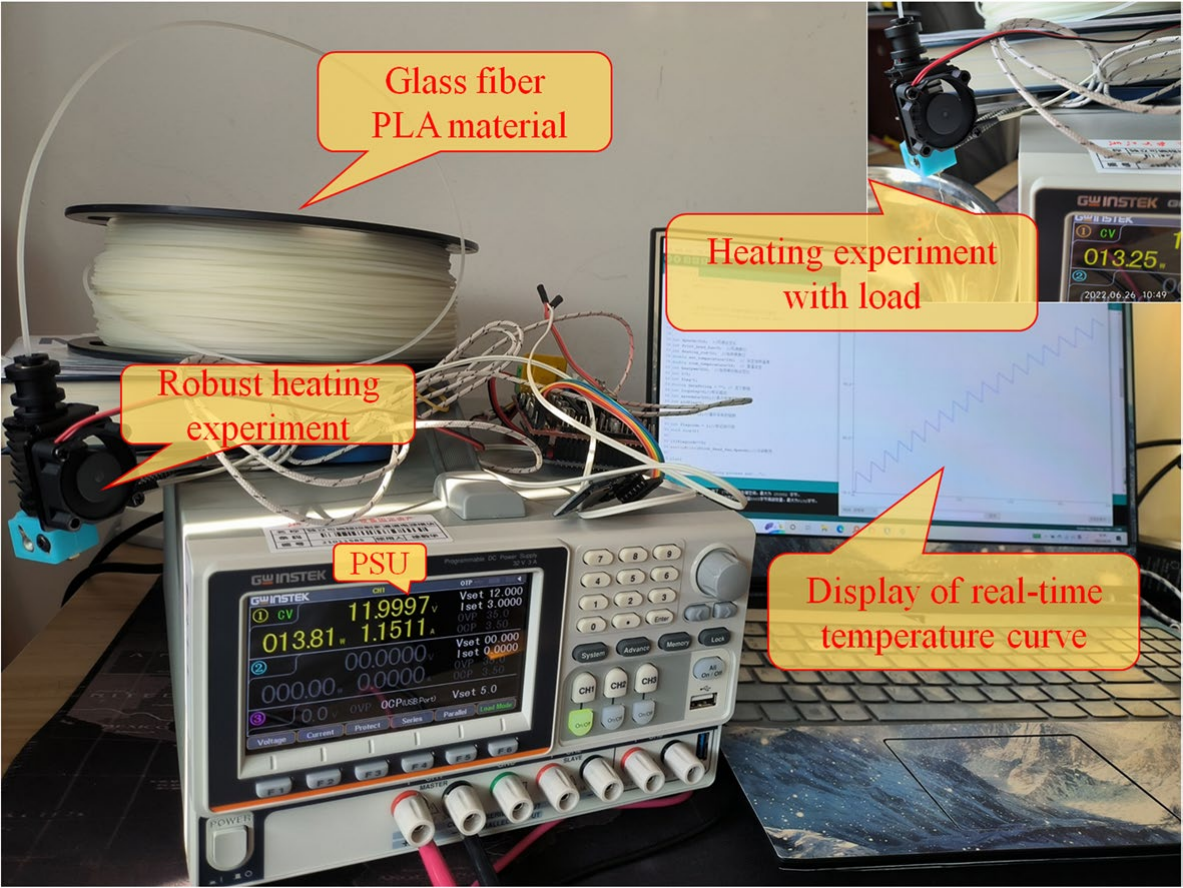
Load test of GF material electrothermal fuzzy heating

To verify the effectiveness of the temperature control strategy on the heating process of the GF PLA (polylactic acid) materials (PLA-GF), temperature control experiments with variable loads were performed. The experimental setup is illustrated in Fig. 4. Double-tube heating has the unique advantages of a heating block and good thermal regulation.

**Fig. 5:**
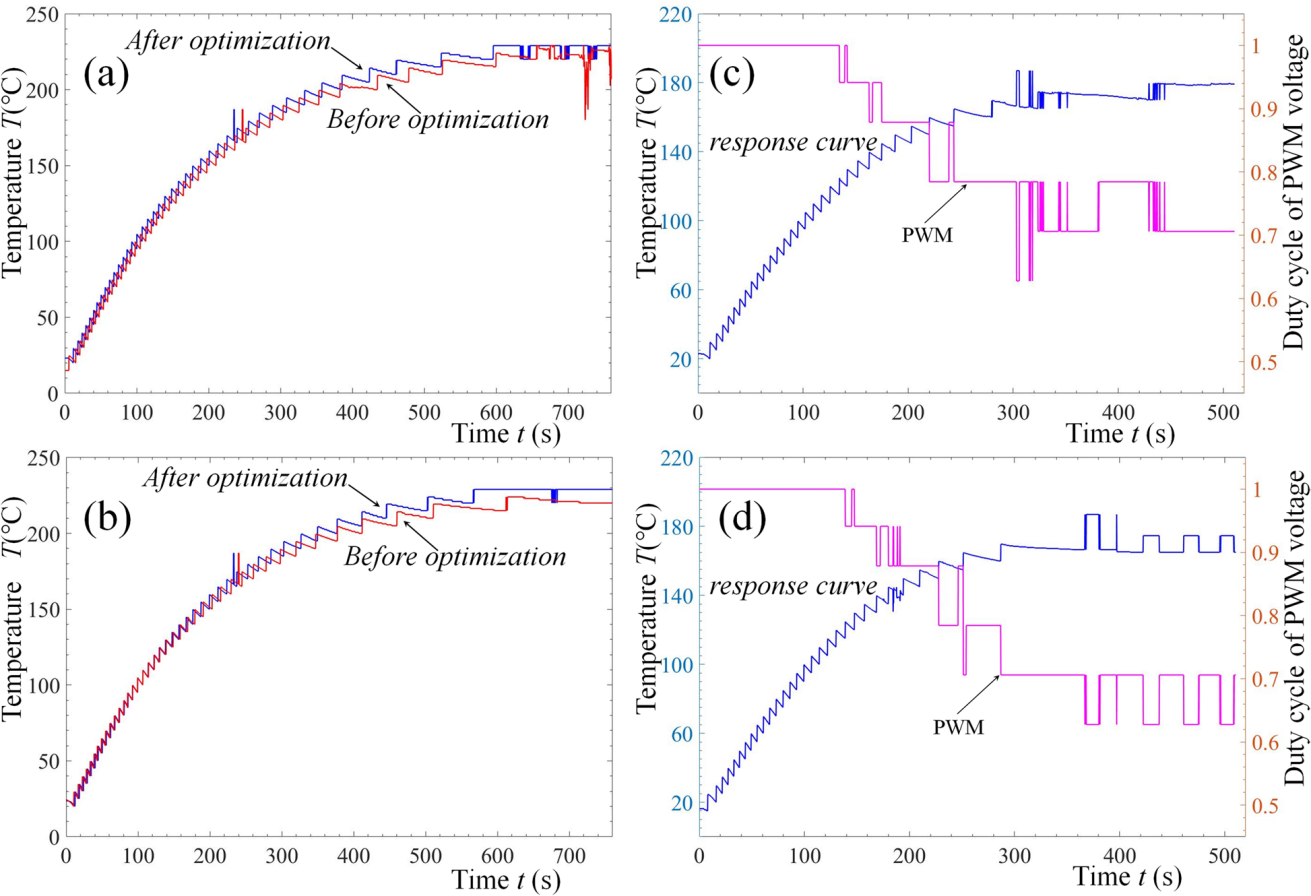
Heating curve of GF PLA of two repeated tests (**a**, **b**) and (**c**, **d**), where the left column is response curve whereas the right is their real-time curve of PWM during thermal regulation

Figure 5 shows the experimental results of the heating temperature control of the PLA-GF material. Figures 5a and c show the experimental data of the two repeated tests. Each group includes curves before and after using the optimization algorithm under the same heating conditions. The PLA-GF material was heated to the melting temperature (230 °C) using a temperature control algorithm before and after improvement. The improved temperature-control algorithm exhibited faster heating and better stability under the same experimental conditions.

Figures 5b and d show the curves of heating the PLA-GF material to the unmelted temperature to demonstrate the change in the output voltage duty cycle (the proportion of high level in one pulse cycle) in fuzzy decision-making, which is realized by pulse width modulation (PWM) of the voltage. They correspond to the optimized heating curves in Figs. 5a and c respectively. From the experimental results, it can be observed that the PWM of the output voltage is large when the difference between the collected data of the sensor and target temperature (180 °C) is large. The PWM of the output voltage begins to decrease when the temperature gradually increases, thus avoiding a control overshoot. When approaching the target temperature, the PWM fluctuates significantly with the temperature difference and its change value. When the temperature is stable, the PWM also tends to be stable and fluctuates within a small range when heating is required. The experimental outcomes were in accordance with the optimization objective of fuzzy decision-making.

**Fig. 6:**
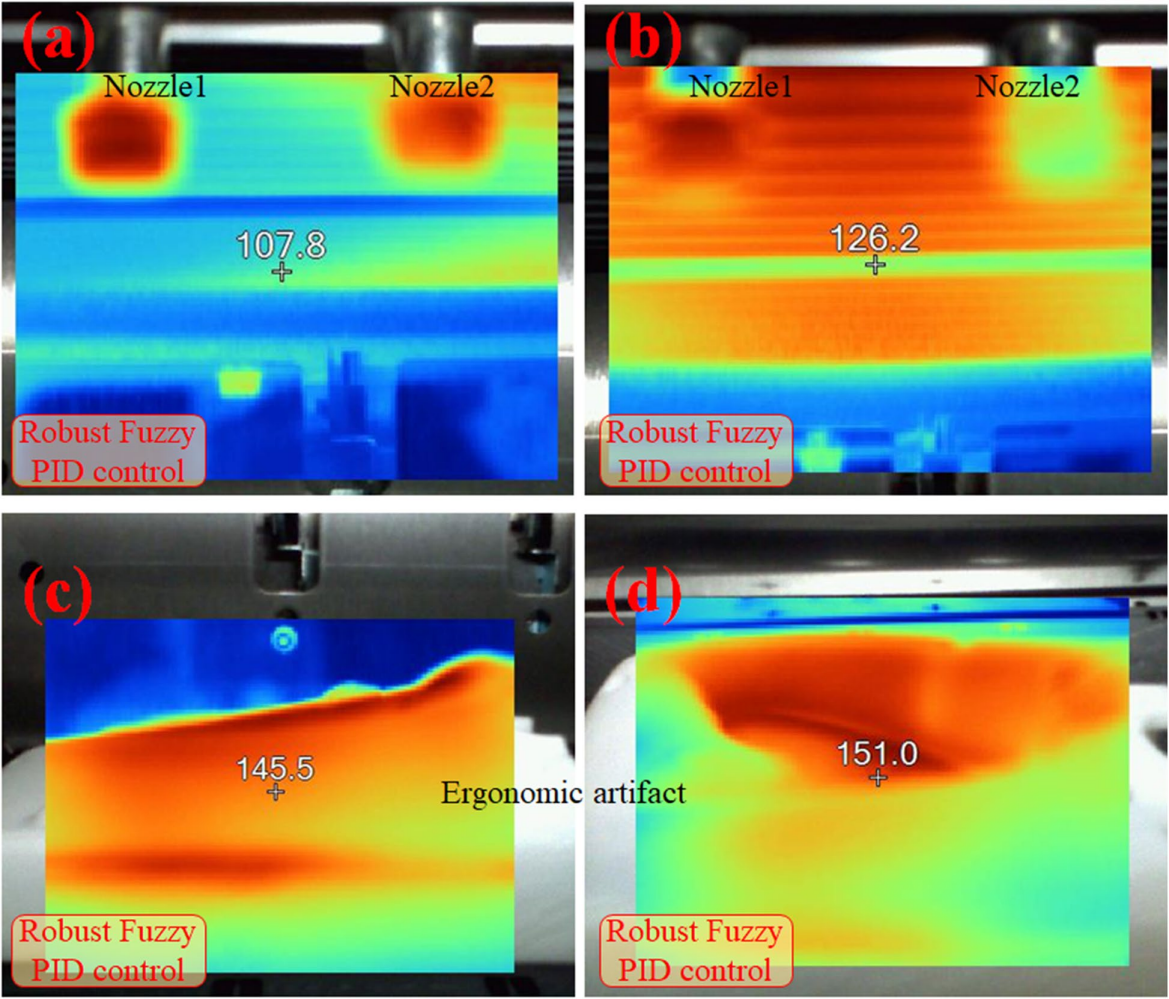
Focused infrared thermographs during the thermoplastic process of a GF composite. The maximum temperatures are 107.8℃, 126.2℃, 145.5℃, 151.0℃, respectively, within almost sealed, high-temperature chamber, in (**a**, **b**, **c**, **d**) upon using fuzzy logic

Infrared thermographs of thermal field measurements obtained using fuzzy logic are shown in Fig. 6. A higher surface temperature indicates remarkable characteristics of the composite material.

## Results and discussion

### Results

#### D functional artifact to be fabricated

The proposed VCDT was implemented on a platform coded in Python 3.7, and all the numerical tests were performed on a PC operating on Windows 10 64-bit.

**Fig. 7:**
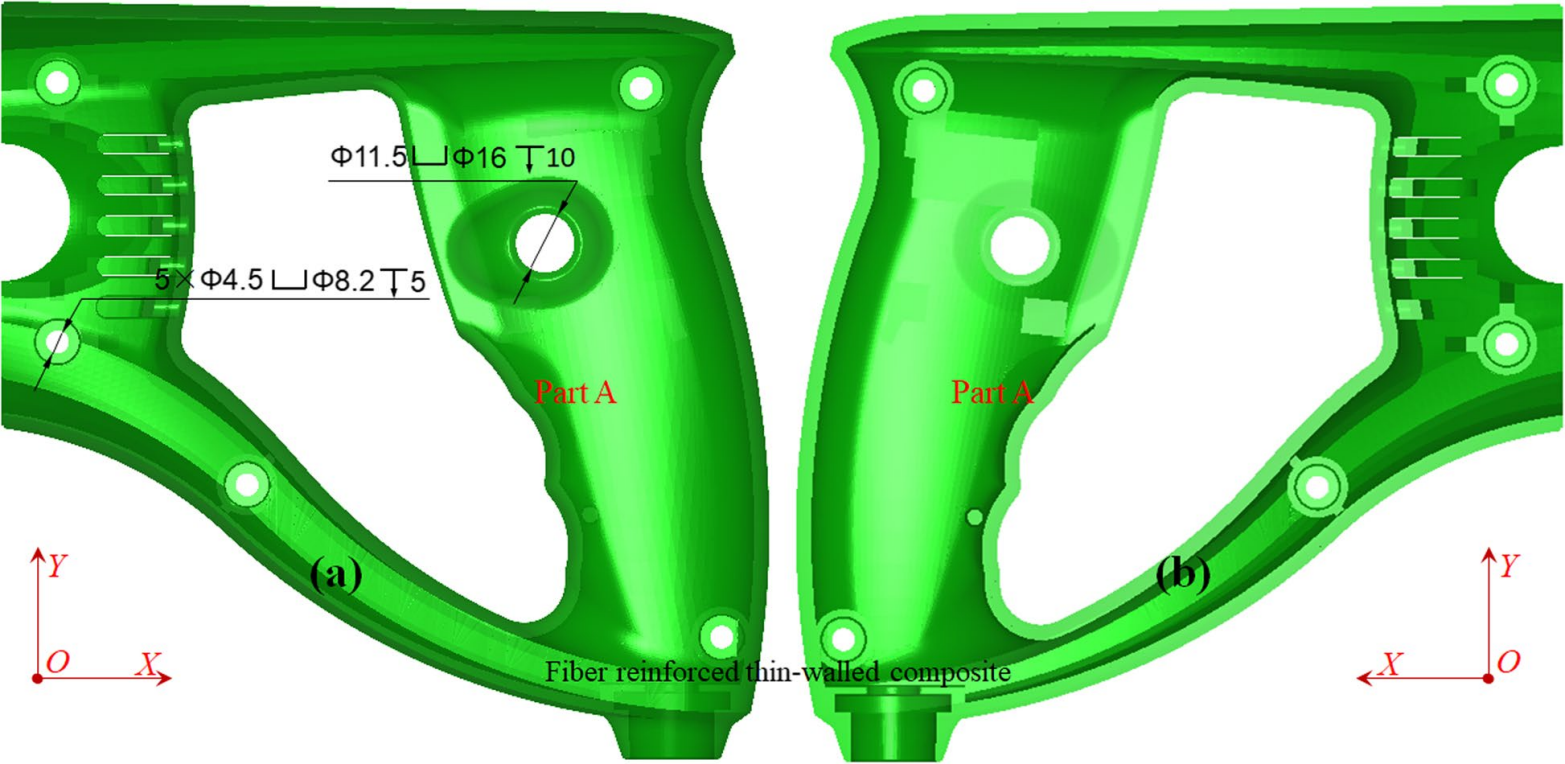
Heavy-duty functional ergonomic half (Part A) handle: **a** main/front view and **b **back view

A slender, thin-walled functional half handle (Fig. 7) was used as a calculation example to verify the previously stated theoretical method. By default, the length unit hereinafter is millimeter (mm).

The overall dimension (${x}_{b}{,y}_{b},{z}_{b}$) of the AABB is equal to (150.9527, 148.9764, 46.5349) with a ratio of 3.2439:3.2014:1. The percentages of the AABB to the center of gravity are (50.4337%, 58.1379%, 42.8853%). The minimum bounding sphere was placed at (148.9994, -33.0316, 8.5189) with a spherical radius of 102.4477. The total surface area ${S}_{object}$ is 46497.5272, the total volume of the enclosed manifold ${V}_{object}$ is 77661.2048, and the specific surface area is 0.7838. The net mass was calculated as 81.5133 g when Acrylonitrile Butadiene Styrene(ABS) was used.

**Fig. 8:**
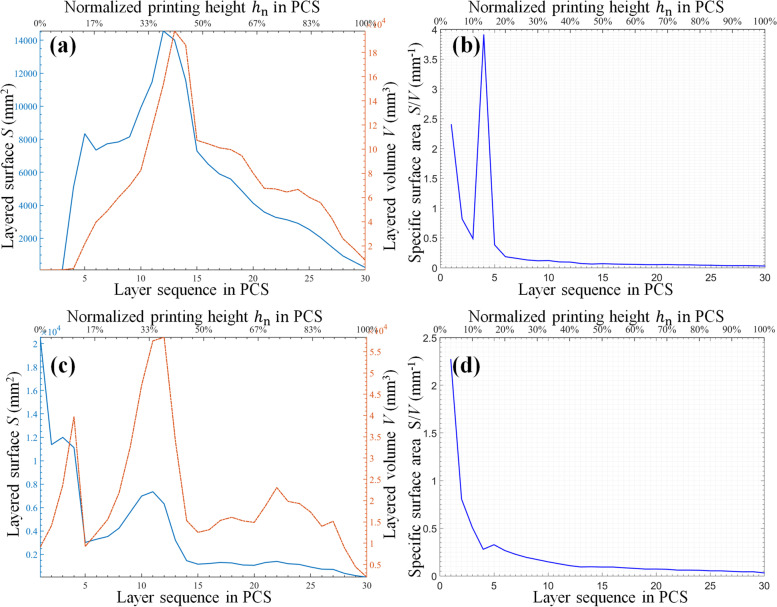
Layered surface and volume along with specific surface area of the functional half handle: **a** and **b** represent the original manifold, whereas **c** and **d** represent the external support

Figure 8 shows the layered surface and volume along with the specific surface area. Table 1 lists the values of the important parameters of the specific surface area and slope corresponding to Figs. 8b and d.

**Table 1:** Specific surface area and slope of functional artifact

Computed parameter		Original manifold (Fig. 8b)	External support (Fig. 8d)
Specific surface area S/V (mm^-1^)	Maximum	2.2705 (at 3.3333%)	2.2705 (at 3.3333%)
	Sum	6.6490	6.6490
	Mean	0.2216	0.2216
	Standard deviation	0.4194	0.4194
	Variance	0.1759	0.1759
Slope of curve	Maximum	-0.0039 (at 14.0000)	8.5350 (at 3.0000)
	Minimum	-7.7724 (at 1.0000)	-10.2791 (at 5.0000)
	Mean	-0.7017 (inclination angle 144.9441°)	-0.4665 (inclination angle 154.9920°)

#### Virtual printing via layered orthogonal projection areas using visual computing digital twins

**Fig. 9:**
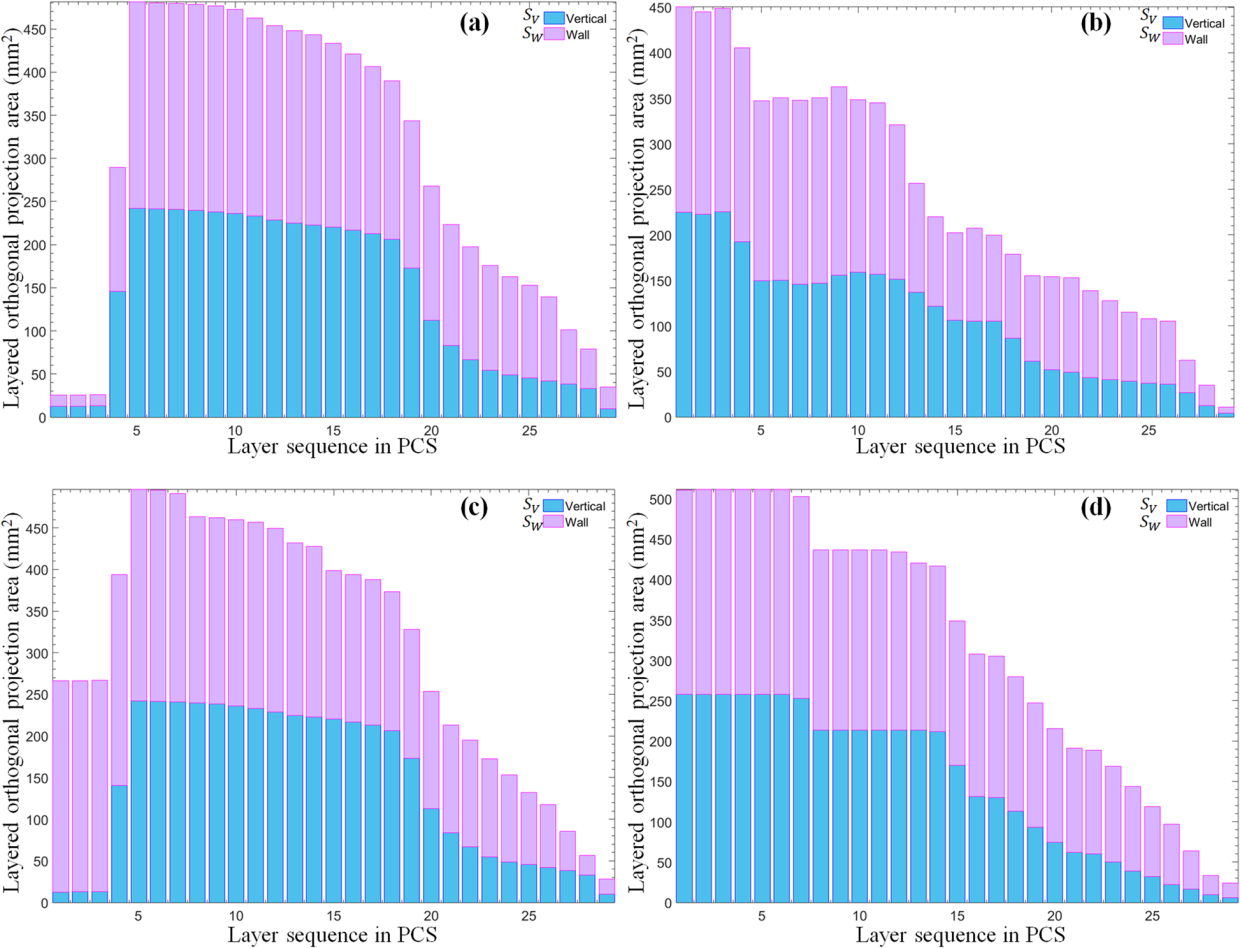
Layered orthogonal projection areas of two typical supports where **a** represents the original manifold and **b** represents the external support manifold of tree type; **c** represents the original manifold and **d** represents the external support manifold of linear type

Figure 9 presents the comparison of the layered orthogonal projection areas of various supports using stacked bars rather than a grouped style. Table 2 lists the corresponding calculation outcomes under different conditions.

**Table 2:** Orthogonal projection area of different manifolds of tree type and linear type

Computed RP parameter		Tree type		Linear type	
		Original manifold	External support	Original manifold	External support
Projection area ${S}_{V}$ in vertical plane (mm^2^)	Maximum	242.0932	225.2717	242.1065	257.5087
	*h*_n_	17.2414%	10.3448%	17.2414%	20.6897%
	Sum	4094.8282	3144.0046	4092.5510	4299.3489
	Mean	141.2010	108.4140	141.1224	148.2534
	Standard deviation	93.4070	66.5183	93.3993	93.6787
	Variance	8724.8710	4424.6831	8723.4231	8775.7021
Projection area ${S}_{W}$ in wall plane (mm^2^)	Maximum	239.0490	225.2443	239.0490	253.6364
	*h*_n_	17.2414%	3.4483%	17.2414%	6.8966%
	Sum	4475.4144	3807.3829	4472.5832	5022.1253
	Mean	154.3246	131.2891	154.2270	173.1767
	Standard deviation	79.4550	66.8469	79.4790	73.6694
	Variance	6313.1020	4468.5063	6316.9064	5427.1736

**Fig. 10:**
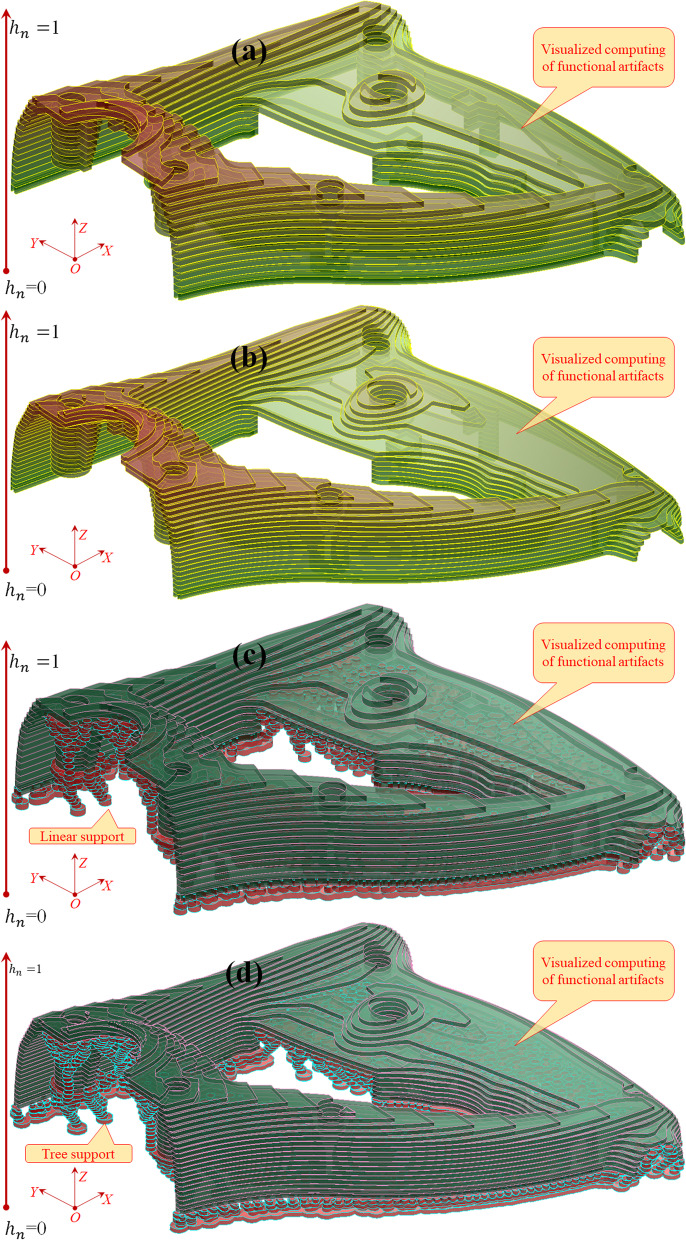
Visualized virtual printing of fiber-reinforced composites using visual computing digital twins, from the front view, where **a** denotes 20 layers without support, **b** denotes 30 layers without support, **c** denotes 20 layers with tree support, and **d** denotes 30 layers with tree support

Figure 10 indicates that visualized virtual 3D printing can be utilized to evaluate the thermoplastic adhesion process.

#### Finite element analysis via transient thermal structure coupling

Finite element analysis was conducted based on transient thermal structure coupling theories using a quasi-linear solution with 8-node 3D thermal solid element. The structural analysis was implemented using the mesh domain decomposition method. Table 3 lists the common physical and chemical properties of ABS and the carbon fiber and GF mixed materials. These parameters were used for the finite element analysis.

**Table 3:** Polymer materials and mixed materials of carbon fiber and GF

Material parameter	ABS	ABS carbon fiber	PAGF
Density ρ (g/cm^3^)	1.05	1.0972	2.4-2.76
Relative dielectric coefficient ${\varepsilon }_{r}$	2.9-3.5	3.2	4-6
Thermal conductivity (W/ (m·K))	0.02-0.046	5.2-6.0	0.7-1.28
Tensile break strength *σ*_*t*_ (MPa)	28.1	37.7	98
Tensile elongation at break *e*_*t*_ (%)	1.5	2.70	3-4
Elastic modulus *E*_*e*_ (GPa)	2.4	3.342	5.7
Flexural break strength *σ*_*f*_ (MPa)	No Break	69	90
Flexural modulus *E*_*f*_ (GPa)	2.22	3.76	5.2
Distortion temperature (°C)	98	101	157
Specific stiffness S_sti_ (N·m/kg)	2.29	3.05	3.0-3.6
Specific strength S_str_ (N·m/kg)	26-76	34.36	12.6-19.7

**Fig. 11:**
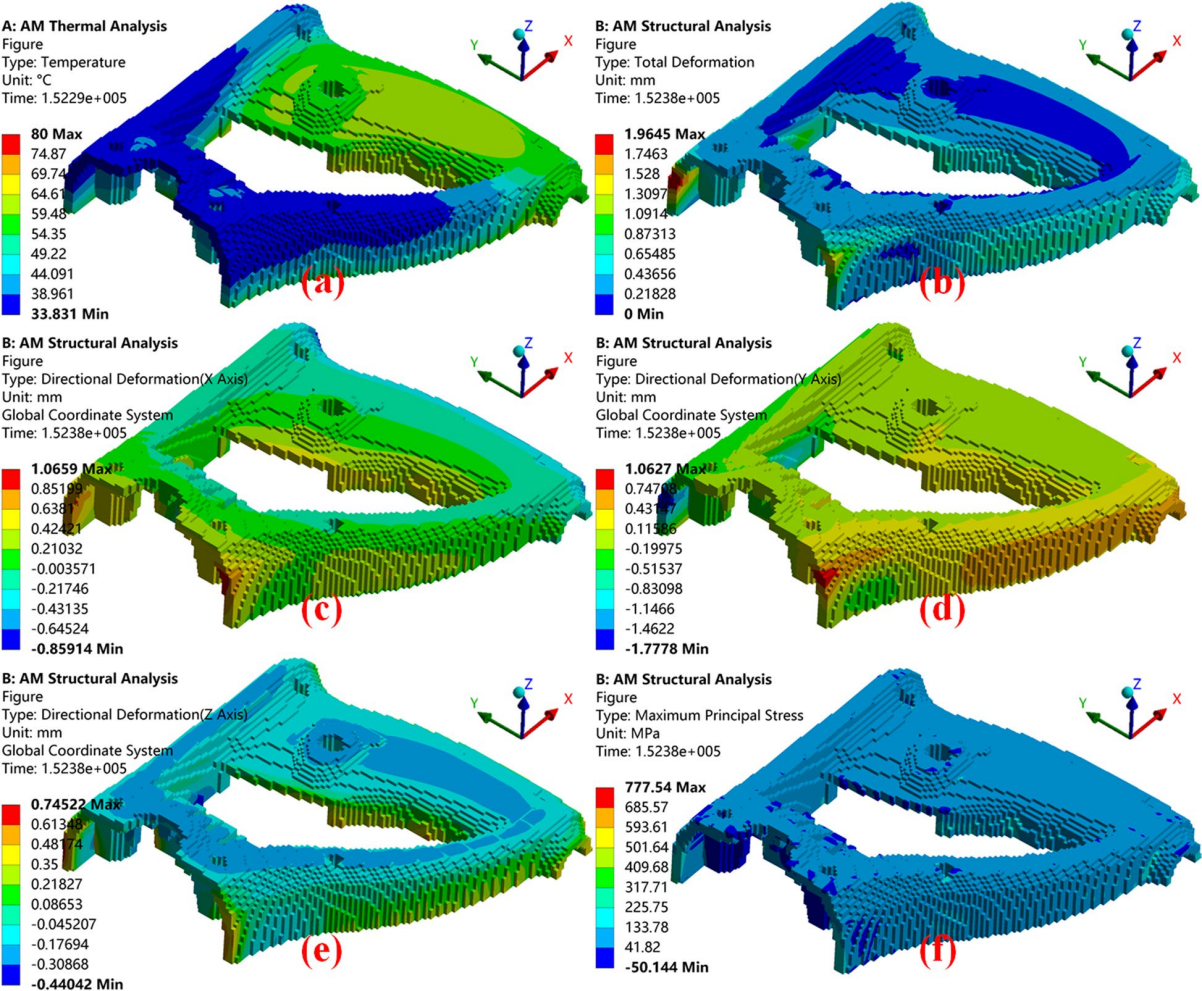
Outcomes of FEA where **a** is the temperature distribution; **b** is the total deformation; and **c**, **d**, and **e** show the directional deformations along the X, Y, and Z axes, respectively; **f** shows the maximum principal stress result

The simulation results of the temperature distribution, total deformation, and directional deformation are shown in Fig. 11. The detailed data are presented in Table 4.

**Table 4:** Detailed outcomes of FEA

FEM outcomes		Value	Relative position ratio
Material temperature (℃) (Fig. 11a)	Maximum	79.496	(0.860, 0.556, 0)
	Minimum	33.831	(0, 0.444, 0.645)
	Average	60.851	/
Total deformation (mm) (Fig. 11b)	Maximum	1.965	(0, 1, 0.484)
	Minimum	0	(0.78, 0.303, 0)
	Average	0.245	/
Directional deformation along X directions, positive, negative and average (mm) (Fig. 11c)	Maximum +	1.066	(0, 0.485, 0.613)
	Maximum –	0.859	(0.960, 0.949, 0.258)
	Average	0.035	/
Directional deformation along Y directions, positive, negative and average (mm) (Fig. 11d)	Maximum +	1.063	(0, 0.444, 0.613)
	Maximum –	1.778	(0, 1, 0.484)
	Average	-0.028	/
Directional deformation along Z directions, positive, negative and average (mm) (Fig. 11e)	Maximum +	0.745	(0.960, 0.960, 0.097)
	Maximum –	0.440	(0.330, 0.859, 0.161)
	Average	-0.060	/
Maximum Principal Stress (MPa) (Fig. 11f)	Maximum +	361.510	(0, 0.980, 0.097)
	Maximum –	-24.642	(0.050, 0.939, 0.677)
	Average	61.754	/

**Fig. 12:**
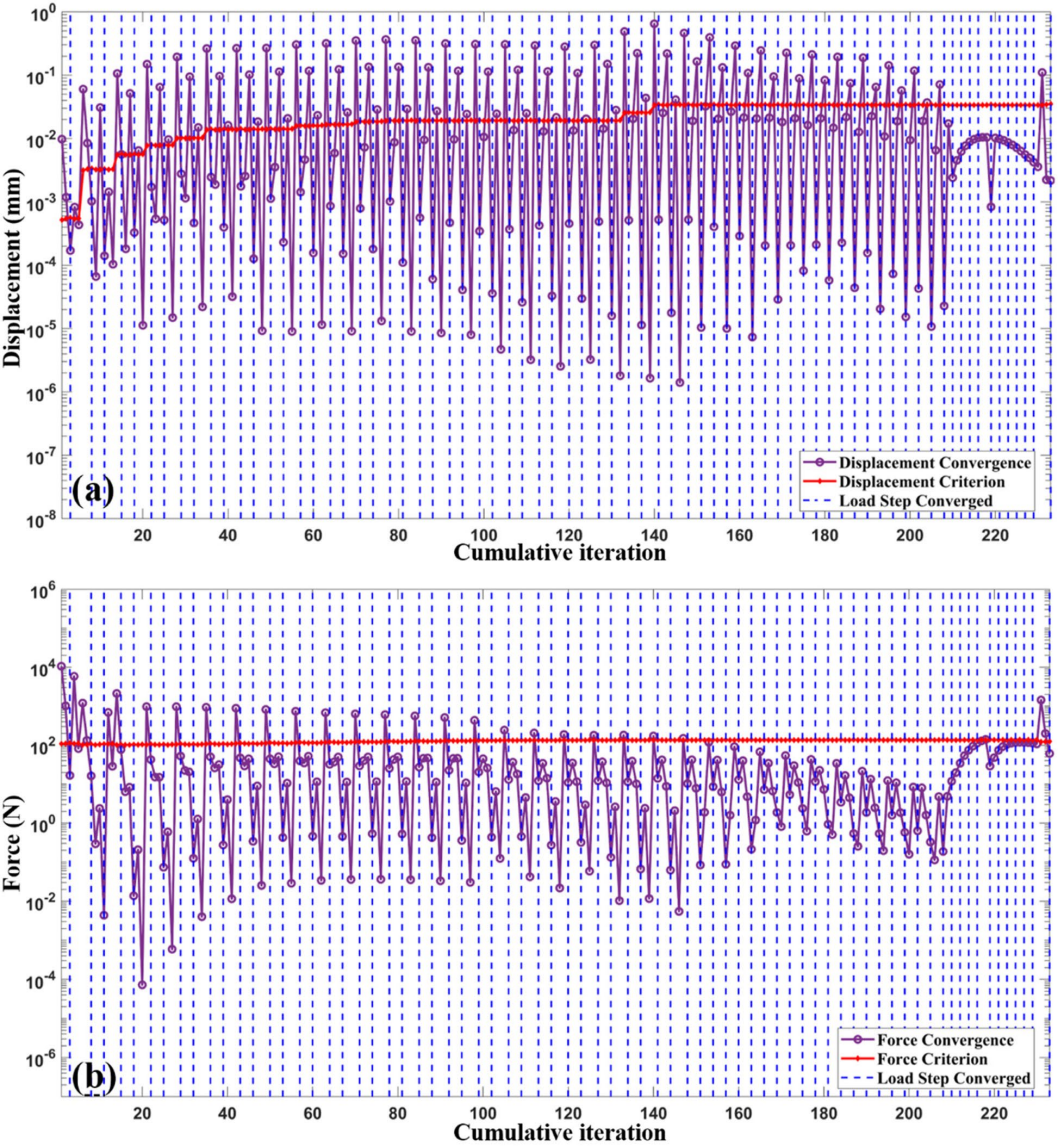
**a** and **b** are displacement and force convergence curves of the FEA simulation process. The magenta lines denote the convergence outcomes of each cumulative iteration step. The red lines represent the criterion values. The blue vertical dotted lines indicate the converged load steps

The convergence outcomes of the cumulative iteration calculations are shown in Fig. 12. The elements were sliced into 52 layers according to their height, and the heat load was applied when the elements of a new layer were active.

#### Experimental test of VCDT

**Fig. 13:**
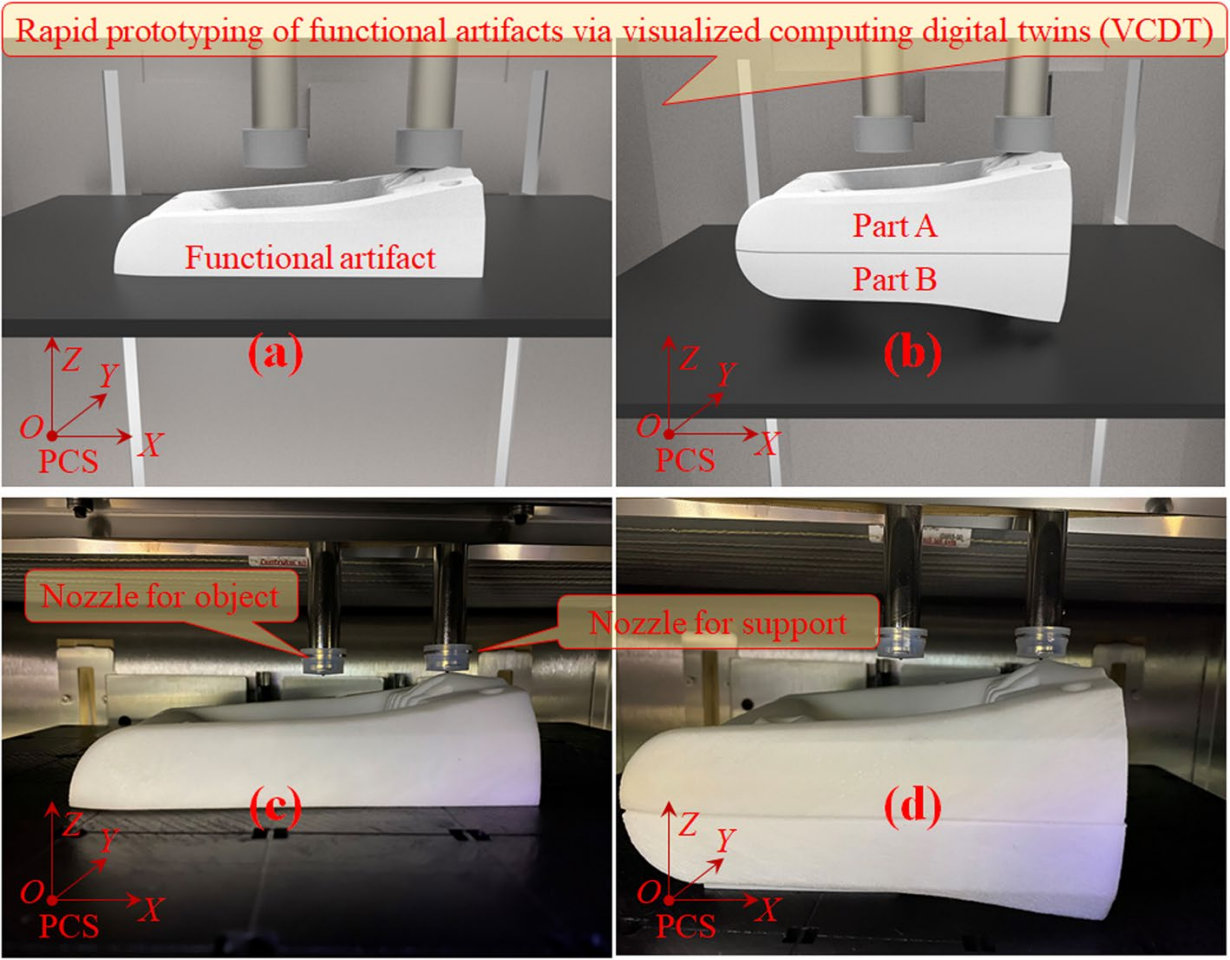
VCDT of RP where **a** and **b** depict the digitally fabricated functional artifacts and **c** and **d** display the physical extrusion-based equipment and functional artifacts

**Fig. 14:**
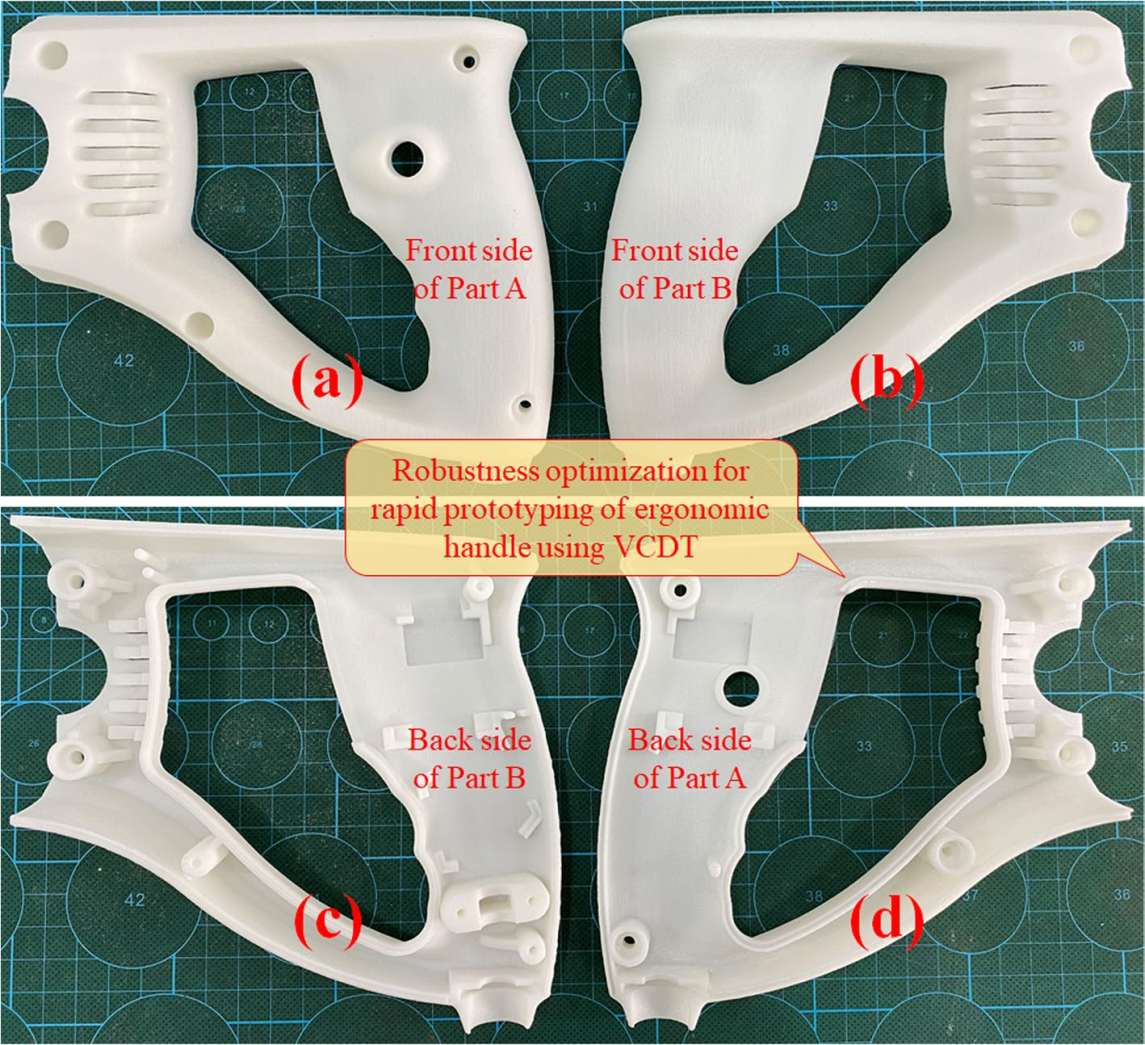
Fabricated ergonomic heavy-duty half handles where **a**, **b**, **c**, and **d** illustrate distinct parallel orthographic views

The stereolithography equipment was an extrusion-based 3D printer operated at an ambient temperature of 25 °C and 55% relative humidity, as depicted in Fig. 13. Digital twinning here can be further divided into visual twinning, functional twinning and mechanism twinning. The layer thickness can be varied from 0.02 mm to 0.3 mm. The resolution precision size in the X/Y direction was 0.1 mm. The fabricated heavy-duty half handles are shown in Fig. 14.

## Conclusions

### (1) A robustness optimization method for RP of functional artifacts based on VCDT was proposed

A generalized multiobjective robustness optimization for RP was first built, where thermal, structural, and multidisciplinary knowledge can be integrated for visualization. The well-known DT was extended to VCDT with a more intuitive computing visualization ability owing to the integration of intelligent multidisciplinary algorithms. This provides a robust design paradigm for a layered RP between the steady balance of electrothermal regulation and manufacturing efficacy under hybrid uncertainties.

### (2) A GA was employed to improve the fuzzy decision-making scheme, which was fed back to the visualization process to obtain a better strategy

A fuzzy decision-making model of RP was established, and the optimal membership function rules were obtained through a GA iteration, which realized a more accurate control scheme. Therefore, the fuzzy decision-making model can be adapted to different material and form requirements and can be customized according to diverse application scenarios.

### (3) A physical experiment regarding layered RP and infrared thermographs was conducted

Infrared thermographs were obtained using thermal field measurements to determine the temperature distribution, which was highly consistent with the DT outcomes. The physical experiment and practice proved that the proposed VCDT provided a robust design paradigm for a layered RP between the steady balance of electrothermal regulation and manufacturing efficacy under complex RP uncertainties.

Future work involves the application of VCDT to more asymmetric artifacts with composite materials via fuzzy heating systems to enhance the universality of the theory in RP for product conceptual design.

## Data Availability

The metadata about robustness optimization of the paper can be shared for availability, in case of publication.

## Abbreviations

RP: Rapid prototyping
VCDT: Visualized computing digital twins
FDM: Fused deposition modeling
GA: Genetic algorithm
DT: Digital twin
AABB: Axis-aligned bounding box
GF: Glass fiber
PID: Proportion integration differentiation
PWM: Pulse width modulation
ABS: Acrylonitrile Butadiene Styrene
